# British versus Continental Spas: A Plea for Home-Made Hydrotherapy

**Published:** 1914-07-11

**Authors:** 


### BRITISH versus CONTINENTAL SPAS.

### A Plea for Home-made Hydrotherapy.

An editorial article in the *Journal of Tropical Medicine and Hygiene* contains some amusing chaff of the pretensions of the European spas, together with some sturdy patriotism in upholding the virtues of British medicinal springs. There is, moreover, a great deal of sound sense in the contentions thus put forward, for much nonsense is talked and written about the fashionable, well-advertised Continental waters. The article naturally takes up the question chiefly from the point of view of those seeking restoration of health and energy after having suffered from some tropical disease; but the guiding principles laid down apply equally well to the diseases of temperate climates, and are worth the attention of thoughtful clinicians.

### Treatment of Tropical Diseases.

It is very truly remarked that a practitioner who is requested to advise a patient concerning a projected visit to a spa usually turns up his reference books and makes a fruitless effort to unravel the rival claims of contending spas to be specific for this or that complaint. From this exercise he emerges more or less confused, because every spa vaunts its efficacy for almost every ailment in the category of disease. Catarrh of every tract of the human body, and of each individual organ in those tracts, is specially mentioned in almost every spa prospectus. Last of all on these lists, complains the *Journal of Tropical Medicine*, stands "tropical diseases." Now, at Continental spas, it need hardly be said, not a single practitioner has any knowledge whatever of tropical medicine of the modern scientific sort. The main idea of most of these practitioners is to deplete all visitors arriving from the tropics, under the idea that they are certain to be suffering from hepatic engorgement and over-generous living. But the average convalescent from malaria and most other tropical diseases is anæmic rather than plethoric, and needs blood-formation rather than severe purging. It is unquestionable that most of the visitors to spas do return benefited, but this result is due as much to the climatic change, the regular life, and the amenities of the place in general, as to the potency of the particular salts which analysts' certificates dilate upon to six places of decimals in their reports upon the character of "the waters."

### Concerts or Cures?

Spas, says the critic, on the Continent are not meant for the treatment of serious diseases, and it behoves really sick folk to avoid them; moreover, no one wants them there. The visitors do not like to see invalids about; they say the sight depresses them. The doctors are only dietetic and balneological specialists, not practitioners of all-round excellence. The entertainments are the primary feature of the attractions; water drinking is merely an addendum, neglected by many. In Britain, on the other hand, entertainments are less considered or are altogether absent; and there are more genuinely sick folk at the spas. The doctors are clinicians, not dietetic experts merely; and they actually treat disease as well as other practitioners do. Also, they are far more likely to know something about tropical hygiene and scientific tropical medicine.

It is hardly to be expected that European spas and those who practise there will take these criticisms lying down; and they are, perhaps, somewhat too sweeping to be completely justified. Nevertheless, there is sufficient truth in them to make them well worth heeding. Certainly, in our judgment, British spas are too much neglected by those in search of healing waters, and foreign resorts stand much higher in the estimation of the lay public than the facts warrant. There is no necessity why anyone for whom spa treatment is advisable should go out of Britain to get it; and if this were generally recognised the results of sending patients to "take the waters" would be much more beneficial than is at present the case.

